# Protein Phosphatases 2A Affects Drug Resistance of *Candida albicans* Biofilm Via ATG Protein Phosphorylation Induction

**DOI:** 10.1016/j.identj.2025.103873

**Published:** 2025-08-30

**Authors:** Jiadi Shen, Chenyu Weng, Shuxian Zhu, Wanjing Chen, Xuening Xiong, Xin Wei

**Affiliations:** a Department of Endodontics, The Affiliated Stomatological Hospital of Nanjing Medical University, Nanjing, China; b State Key Laboratory Cultivation Base of Research, Prevention and Treatment for Oral Diseases, Nanjing, China

**Keywords:** *Candida albicans*, Protein phosphatases 2A, Autophagy, Biofilm formation, Drug resistance

## Abstract

**Objectives:**

*Candida albicans* (*C. albicans*) biofilms are well-known to be resistantto various antifungal agents. Autophagy is crucial for adapting to changes in nutrition conditions. Protein phosphatase 2A (PP2A) is prominent in regulating physiological processes, possibly related to autophagy-related (ATG) protein phosphorylation. This study hypothesizes that PP2A affects biofilm formation and drug resistance of *C. albicans* via autophagy induction.

**Materials and methods:**

The expressions of PP2A catalytic subunit coding gene *PPH21* were compared. The mutant strain (*pph21*Δ/Δ) was constructed and the biofilm was treated with autophagy activator (rapamycin, Rap). The biofilm formation, drug susceptibility and oxidative stress levels were examined. The autophagic activity was detected, along with the autophagosomes observed. The therapeutic efficacy of the antifungal agents was estimated on the mice model of *C. albicans* oral infection.

**Results:**

*PPH21* was associated with *C. albicans* biofilm formation and drug resistance. Autophagy activation by rapamycin can induce increased autophagy levels, while it was hindered in *pph21*Δ/Δ. Besides, the protein levels of Atg13 and Atg1 were significantly down-regulated in *pph21*Δ/Δ+Rap (*P* < .01), along with its decreased regulatory capacity to oxidative stress. Autophagy activation can promote biofilm formation and improve drug resistance, while the absence of *PPH21* may prevent the enhancement of drug resistance. Autophagy activation reduced the efficacy of antifungal agents in treating oral *C. albicans* infection in mice, among which *pph21*Δ/Δ presented better therapeutic effects.

**Conclusion:**

PP2A is important in the autophagy induction of *C. albicans* by participating in Atg13 phosphorylation, followed by Atg1 activation, further affecting its biofilm formation and drug resistance.

**Clinical relevance:**

PP2A-induced autophagy may be a potential regulatory mechanism of *C. albicans* drug resistance. This appears to be a promising therapeutic strategy for managing *C. albicans*-related infectious diseases.

## Introduction

*Candida albicans* (*C. albicans*) is one of the common opportunistic fungal pathogens, which is generally found in multiple sites of healthy persons, such as gastrointestinal, respiratory, and genitourinary tracts.^1^ It can induce various infections in immunocompromised or immunologically deficient individuals, which may turn from local opportunistic or commensal infections of the mouth to stemic invasive candidiasis.^2^^,^^3^ In recent years, with a rapid increase in the incidence of candidiasis, as well as its resistance to traditional antifungals, relevant treatment of fungal infections remains considerably challenging and generates high healthcare costs worldwide.^4^^,^^5^

A major factor associated with the virulence of *C. albicans* is their capacity to form biofilms, which occur progressively and mainly consist of yeast cells, pseudohyphae, and hyphae.^6^^,^^7^ *C. albicans* biofilms, as highly organized attached microbial communities, are inherently resistant to commonly used antifungal drugs, making biofilm-based infections a major clinical challenge.^8^^,^^9^ Currently, there are limited antifungal drugs available in clinical practice, including azoles, echinocandins, and polyenes, the efficacy of these drugs is compromised by the emergence and development of drug-resistant *C. albicans* strains. Accordingly, it is necessary to explore the mechanism of biofilm formation and drug resistance in *C. albicans*.

Autophagy is a highly conserved catabolic mechanism that occurs in response to changes during starvation for nitrogen or carbon.^10^ Target of rapamycin (TOR), as a major regulator of nutrient signalling, can form 2 functionally distinct complexes: TOR complex 1 (TORC1) and TOR complex 2 (TORC2).^11^ TORC1, a nutrient-responsive protein kinase, can regulate metabolism and cellular growth, along with the suppression of catabolic processes like autophagy.^12^ The activity of TORC1 plays a crucial role in regulating autophagy induction,^13^ where the active TORC1 inhibits autophagy induction by phosphorylating the Atg13 subunit of the Atg1 complex under nutrient-sufficient conditions.^14^ Consequently, inhibition of TORC1 is a prerequisite for controlling autophagy.^15^^,^^16^ TORC1 is inactivated under starvation or rapamycin treatment, which contributes to Atg13 dephosphorylation, while Atg13 is phosphorylated under nutrient-replete conditions.^17^ The TORC1-Atg1 signalling axis is considered to be conserved throughout most eukaryotes to regulate autophagy, in which Atg1 and Atg13 are critical components required for autophagy induction and autophagosome formation.^18^^,^^19^ Although dephosphorylation of Atg13 was reported to be one of the initial processes of autophagy in cells,^20^ whether it is sufficient to induce autophagy in fungi remains to be elucidated.

Various phosphatases can dephosphorylate Atg13, among which some protein kinases have been proven to be associated with Atg13 phosphorylation, such as protein kinase A.^21^ Protein phosphatase 2A (PP2A), a member of the serine/threonine (Ser/Thr) phosphatase family, appears to be responsible for regulating protein phosphorylation.^22^ In yeast, PP2A is composed of 3 distinct subunits: A subunit (structural subunit, encoded by *TPD3*, B subunit (regulatory subunit, encoded by *CDC55* and *RTS1*, and C subunit (catalytic subunit, encoded by *PPH21* and *PPH22*).^23^ The genome of *C. albicans* contains only 1 PP2A catalytic subunit encoded by *PPH21*,^24^ which can be speculated that PP2A may be involved in autophagy by affecting Atg13 dephosphorylation in *C. albicans*. In addition, it remains to be addressed whether *PPH21* influences PP2A activity, together with Atg13 dephosphorylation, thereby affecting the autophagy induction of *C. albicans*. In this study, the role of PP2A in autophagy induction of *C. albicans* was evaluated, as well as its effects on biofilm formation and drug resistance*.*

## Materials and methods

### Strains and growth conditions

*C. albicans* standard strain *SC5314* was purchased from the American Type Culture Collection (ATCC, Manassas, VA, USA). *C. albicans* strain *SN152* and the plasmids (*pSN52* and *pSN40*) were provided by the State Key Laboratory of Pharmacy Genetic Engineering, Second Military Medical University, Shanghai, China (Table 1). The protocol for inducing the drug-resistant strain was based on previous research.^25^ The activity of fluconazole against planktonic forms of *C. albicans SC5314* was measured by determining the minimal inhibitory concentration (MIC), following the Clinical and Laboratory Standards Institute (CLSI) guidelines.^26^ When the fluconazole MIC reached or exceeded 64 μg/mL (MIC ≥ 64 μg/mL), the strain was considered fluconazole resistant (ie, drug-resistant strain in this study). All strains were stored as frozen stock with glycerol at −80 °C until use.

**Table 1:** *C. albicans* strains and plasmids used in this study.

	Genotype and description	Reference
Strains		
*SC5314*	Wild-type clinical isolate	American Type Culture Collection (ATCC)
*SN152*	*arg4/arg4 leu2/leu2 his1/his1 URA3/ura3::imm434* *IRO1/iro1::imm434*	^28^
*pph21*Δ/Δ	*arg4/arg4 leu2/leu2 his1/his1 URA3/ura3::imm434* *IRO1/iro1::imm434* *pph21::C.d.LEU2/pph21::C.d.HIS1*	this study
Plasmids		
*pSN52*	With *HIS1* marker	^28^
*pSN40*	With *LEU2* marker	^28^

The mutant strain (*pph21*Δ/Δ) was generated from *C. albicans SN152* using the *HIS-LEU-ARG* knockout strategy.^27^ Selective mediums (SD-his, SD-leu, and SD-his-leu) were employed for positive selection during the construction of the mutant strain. The Yeast Extract Peptone Dextrose (YPD) medium (2% peptone, 1% yeast extract, and 2% glucose) was routinely prepared for *C. albicans* recovery and incubation. *C. albicans* strains and plasmids used in this study were listed in Table 1.

### Preparation of agents

All the agents (rapamycin, fluconazole, itraconazole, amphotericin B, and terbinafine were purchased from APExBIO Technology (Houston, TX, USA). The stock solutions of these agents aforementioned were dissolved in dimethyl sulfoxide (DMSO) and stored at −20 °C until use. Besides, *C. albicans SN152* were treated with different concentrations of rapamycin (Rap, an inhibitor of the target of rapamycin, TOR, 0-400 nM) and incubated at 37 °C for 24 hours. Subsequently, the metabolic activity of the biofilm was measured by XTT [2,3-bis-(2-methoxy-4-nitro-5-sulfophenyl)-2H-tetrazolium-5 -carboxanilide] reduction assay (APExBIO Technology LLC, Houston, TX, USA).^29^ In this study, the concentration of 0% inhibition of biofilm metabolic activity compared with the wild type control (*SN152*) biofilm without rapamycin treatment, was selected as the reference concentration for subsequent experiments.

### Biofilm formation

*SN152* and *pph21*Δ/Δ strains were cultivated in YPD medium overnight at 30 °C in a shaking incubator (200 rpm) to reach the logarithmic growth phase.^30^ The cells were collected, followed by washed with phosphate-buffered saline (PBS) and resuspended in Roswell Park Memorial Institute (RPMI) 1640 medium (Gibco Ltd, Paisley, UK) at a concentration of 1 × 10^6^ cells/mL, as measured at an optical density of 0.1 at 600 nm (OD_600_). The suspensions were inoculated on the surface of 96-well plates or culture dishes (Thermo Fisher Scientific Inc., Waltham, MA, USA) and incubated at 37 °C for biofilm formation. Then, *C. albicans* biofilm was treated with rapamycin for 24 hours, and washed with sterile PBS for subsequent experiments.

### Biofilm development assay

The biofilm development assay was evaluated by calculating the reduction of XTT by adopting the protocol reported earlier.^31^ XTT was prepared at a final concentration of 1 mg/mL in PBS and then stored at −80 °C until use. A 0.4 mM menadione solution (Sigma-Aldrich, St Louis, MO, USA) was mixed with the XTT solution before use. Briefly, after biofilm formation, 96-well plates were washed 3 times with sterile PBS. XTT was added to each well and incubated in the dark for 2 hours at 37 °C. Absorbance was recorded at 490 nm using a microplate reader (SpectraMax M2, Molecular Devices, CA, USA).

### Scanning electronic microscopy (SEM)

The biofilm was grown on sterile coverslips pre-treated with poly-l-lysine measuring 8 mm in diameter and 2 mm in thickness. Then, the formed biofilm was fixed in 2.5% glutaraldehyde overnight at 4 °C. After rinsing with sterile PBS, the samples were dehydrated by alcohol solutions with increasing concentrations of ethanol and dried, followed by sputter-coated with gold (10 ± 2 nm). The morphological changes were observed by scanning electron microscopy (SEM; JEOL JSM-7900F, Japan) operated at 2 kV.

### Antifungal susceptibility testing

The susceptibility of biofilm to antifungal agents was measured using XTT reduction assay. Antifungal agents used in this study were prepared in serial 2-fold dilutions following the M27-A4 document of the Clinical and Laboratory Standards Institute (CLSI, USA) (2017). First, *C. albicans* biofilm was grown on the 96-well plates and subsequently incubated for 6, 12, 24, and 48 hours at 37 °C, respectively. Thereafter, 2-fold serial dilutions of the antifungal agents were added to the biofilm and the plates were incubated for an additional 24 hours at 37 °C. The wells without agent treatment were set as the control group. Subsequently, the metabolic activity of the biofilm was determined by XTT assay as described above. Then, SMIC_50_ (Sessile minimum inhibitory concentration 50%) was calculated, where SMIC_50_ was defined as the antifungal concentration that inhibits 50 % of biofilm metabolic activity compared with the drug-free control group.^32^

### Reactive oxygen species (ROS)

The ROS level was evaluated by the oxidant-sensitive agent 2’,7’-dichlorofluorescin diacetate (DCFH-DA).^33^ After non-adherent cells were removed, the biofilm was incubated with DCFH-DA (Beyotime Biotechnology, Shanghai, China) for 1 hour at 37 °C in the dark, and then the biofilm was washed to remove residual DCFH-DA fully. The fluorescence images were obtained by a fluorescence microscope (Leica DMI3000B, Wetzlar, Germany). For quantitative assays, the fluorescence intensity (excitation, 488 nm; emission, 520 nm) was recorded using a fluorescence spectrophotometer (SpectraMax M2, Molecular Devices, CA, USA), and the relative ROS expression was calculated as fluorescence intensity divided by the cell number.

### Mitochondrial membrane potential (MMP)

Mitochondrial membrane potential (MMP, ΔΨ) is one of the important pointers for indicating the structure and function of mitochondria, which can be evaluated through JC-1 assay.^34^ JC-1 (a cationic dye) is usually localized in mitochondria and serves as a reliable fluorescent probe to assess the mitochondrial ΔΨ,^35^ which exhibits 2 fluorescence emissions: (1) red fluorescent J-aggregates (emission maximum at 590 nm); (2) green fluorescent J-monomers (emission maximum at 529 nm) indicating a decrease in ΔΨ.^36^ The biofilm was stained with JC-1 (Beyotime Biotechnology, Shanghai, China) at 37 °C for 30 minutes in the dark. The fluorescence images were observed by a fluorescence microscope (Leica DMI3000B, Wetzlar, Germany), while changes in MMP of planktonic cells were also analyzed using a flow cytometer (BD Biosciences, NJ, USA), and then the relative ratio of the red/green fluorescence was calculated.

### Alkaline phosphatase (ALP) activity

Alkaline phosphatase (ALP) activity assay can be used to indicate the occurrence of autophagy.^37^ The biofilm was harvested and quantitatively assessed with an alkaline phosphatase assay kit (Beyotime Biotechnology, Shanghai, China). Absorbance was recorded at 405 nm using a microplate reader (SpectraMax M2, Molecular Devices, CA, USA). The results were normalized to the total protein content and presented as nanomoles of p-nitrophenol produced per min per mg protein (nmol/min/mg protein).

### Acridine orange (AO) staining assay

Acridine orange (AO) staining can be employed to observe autophagy in yeast,^38^ which emits red fluorescence in acidic autophagic vacuole vesicles, but green fluoresces in the cytoplasm and nucleus. AO staining was performed using an acridine orange detection kit (Solarbio, Beijing, China). Then, the biofilm was incubated with the AO solution respectively, and observed under a fluorescence microscope. Besides, we measured the percentage of AO-positive cells with a flow cytometer (BD Biosciences, NJ, USA).

### Transmission electron microscopy (TEM)

Transmission electron microscopy (TEM) was used for morphological observation of the autophagosomes in biofilm. The biofilm was cultured as mentioned above (the section 'Materials and methods 2.3–Biofilm formation'), harvested, washed with PBS, and fixed in 2.5% glutaraldehyde overnight at 4 °C, followed by post-fixing with 1% osmium tetroxide solution for 1 hour. Then, the samples were dehydrated in an ethanol series (30, 50, 70, 80, 90, and 100%) and embedded with epoxy resin (Epon-812). Ultra-thin sections were cut and stained with 1% uranyl acetate for 20 minutes, followed by lead citrate for 5 minutes. Eventually, the images were detected by TEM (Carl Zeiss, Oberkochen, Germany).

### Real-time quantitative PCR (RT-*q*PCR)

The biofilm for RT-*q*PCR analysis was formed on the surface of culture dishes, referring to the section 'Materials and methods 2.3–Biofilm formation'. The samples were harvested and immediately frozen in liquid nitrogen. Total RNA extraction of *C. albicans* biofilm was performed using an RNA extraction Kit (Vazyme, Nanjing, China), and the integrity of the RNA from each group was assessed by nanodrop (NanoDrop One, Thermo Fisher Scientific Inc., Waltham, MA, USA). The extracted RNA was subjected to reverse transcription into cDNA using HiScript II RT SuperMix (Vazyme, Nanjing, China) according to the manufacturer’s instructions. Expression levels of the target genes and internal reference gene (*β-actin*) were assessed by RT-*q*PCR, which were performed on a QuantStudio™ 7 Flex system (Thermo Fisher Scientific Inc., Waltham, MA, USA) using ChamQ Universal SYBR *q*PCR Master Mix (Vazyme, Nanjing, China). The relative quantification was calculated using the 2^-ΔΔCt^ method.^39^ Target gene expression was normalized against *β-actin* transcript levels. The primers and sequences were listed in Table 2, which were designed by Shanghai General BioTech Co., Ltd.

**Table 2:** Primers and sequence used in the present study.

Primer name	Sequence (5′ → 3′)
*Atg1*-F	TACAACCCAACTGAGCGGAT
*Atg1*-R	GTAGTGGGTGATGGGCTTCT
*Atg13*-F	GCCAAGACTACGGGGTATGA
*Atg13*-R	AAGCATTGGAATTGCGTCGA
*Atg17*-F	TTCAACGCCTTCCAGCAA
*Atg17*-R	TGGTTTGATCTCTGGCATTGA
*Atg27*-F	ACTCCAACAGCTATCTCGCA
*Atg27*-R	TATAACGTCGCCAACCCTG
*β-actin*-F	GACCAAGAAGACATCAAGGTATCAT
*β-actin*-R	GTGTTCAATTGGGTATCTCAAG

### Western blotting

The *SN152* and *pph21*Δ/Δ biofilm for Western blotting was formed on the surface of culture dishes, referring to the section 'Materials and methods 2.3–Biofilm formation', and then the samples were harvested. Total proteins of the biofilm were extracted based on an immunoprecipitation protocol as previously described.^40^ Then, the protein concentration was determined by a BCA protein assay kit (Beyotime Biotechnology, Shanghai, China). Total protein samples from different groups were first electrophoresed through 10% SDS-PAGE gels and then transferred to polyvinylidene fluoride (PVDF) membranes (Millipore, Billerica, MA, USA). After being blocked with 5% skimmed milk in TBST (2.41% Tris, 8% NaCl, 1% Tween-20) at room temperature for 1 hour, membranes were incubated with primary antibodies (Atg1, Atg13, Atg17, and Atg27, 1:500 dilution) (Dia-An Biotech, Wuhan, China) overnight at 4 °C, followed by incubated with corresponding HRP-conjugated secondary antibodies (1:10000 dilution) for 1 hour. The target bands were visualized with a chemiluminescence system (Merck & Co., Inc., Kenilworth, NJ, USA), where GAPDH (Bioworld, Minneapolis, MN, USA) was used as a reference protein. 7.5% acrylamide gels were used for SDS-PAGE to detect the phosphorylation statuses of Atg13 and Atg1. Relative protein amounts were analysed using Image J software.

### Murine model of oral candidiasis

Animal experiments were approved by the Institutional Animal Care and Use Committee of Nanjing Medical University (IACUC-2307018). Six-week-old female ICR mice were obtained from the Medical Laboratory Animal Center of Nanjing Medical University. The construction of the murine oral candidiasis model was performed according to the protocol described by Takakura et al.^41^ To improve the sensitivity of mice to *C. albicans*, the mice were immunosuppressed with 10 g/L prednisolone (Aladdin, Shanghai, China) 1 day before and 3 days after the inoculation of *C. albicans*. 0.83 g/L Tetracycline hydrochloride (Aladdin, Shanghai, China) administered in drinking water was given to the mice daily until the end of the experiment. Mice were anesthetized by intramuscular injection of 50 μL 2 g/L chlorpromazine (Aladdin, Shanghai, China) and then were inoculated by the cotton swabs fully soaked with the *C. albicans* cell suspension at a concentration of 2 × 10^8^ CFU/mL over the entire oral cavity (including the buccal mucosa, tongue, soft palate, and other oral mucosa surfaces) to produce oral infections. Due to poor absorption through the gut of polyenes, this study chose topical application, a principal method of amphotericin B (Amb) in oral candidiasis. Antifungal agent (Amb) was applied orally and topically on the tongue surface after *C. albicans* inoculation.

At the indicated time postinfection (on the third day of infection based on a previous study),^42^ mice were sacrificed by cervical vertebra dislocation. Murine tongue lesions were detected by macroscopic observation and weight loss was also counted. Viable *C. albicans* cells were calculated as colony-forming unit (CFU) in the murine oral cavity and feces to evaluate the progression of the infection following the protocols of Takakura et al using microbiological evaluation.^41^ The cotton swabs were used to wipe the entire oral cavity of the infected mice and then immersed in a tube containing sterile saline. *C. albicans* cells were resuspended, mixed, serially diluted, plated on SDA agar plates, and incubated at 37 °C for 48 hours, and then the CFU of *C. albicans* was calculated. Besides, the tongues were removed, formalin-fixed, and paraffin-embedded. Tissues were sectioned at 5 μm thickness and stained with Periodic Acid-Schiff (PAS), followed by observation under a light microscope (Leica DM4000, Wetzlar, Germany) for histopathological examination.

### Statistical analysis

All quantitative experiments were performed in triplicate. The data were presented as the mean ± standard deviations (SD). One-way ANOVA was performed to compare the statistical significance of differences after validating the equal variance assumption of data. Within-group variation was assessed via the mean square error (MSE), derived from the within-groups sum of squares (SSW). The significance of overall differences between groups was then determined by evaluating the F-statistic. Post-hoc comparisons were conducted using Tukey's Honestly Significant Difference (HSD) test. All statistical tests were analysed using SPSS 22.0 statistical software (SPSS Inc., Chicago, IL). Differences were considered statistically significant for *P* values of <.05.

## Results

### *PPH21* was related to biofilm formation and drug resistance of *C. albicans*

**Fig. 1:**
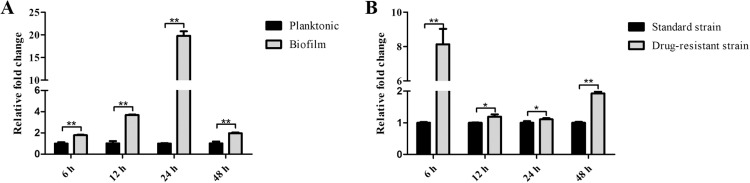
The gene expressions of *PPH21* in *C. albicans* at different phases (6, 12, 24, and 48 h). A, Comparison between *C. albicans SC5314* planktonic cells and biofilm; B, Comparison between the standard strain (*C. albicans SC5314*) and the drug-resistant strain (fluconazole-resistant strain) biofilm. *P < .05; ^⁎⁎^*P* < .01.

To determine whether *PPH21* was involved in the biofilm formation and drug resistance of *C. albicans*, RT-*q*PCR analysis was performed and showed that *PPH21* expressed significantly higher levels at different phases (6, 12, 24, and 48 h) in biofilm than in planktonic cells (*P* < .01) (Figure 1A); moreover, its expression was also significantly upregulated in drug-resistant strain compared with those in standard strain (*P* < .05) (Figure 1B).

### Autophagy activation by rapamycin affects the biofilm formation of *C. albicans*

**Fig. 2:**
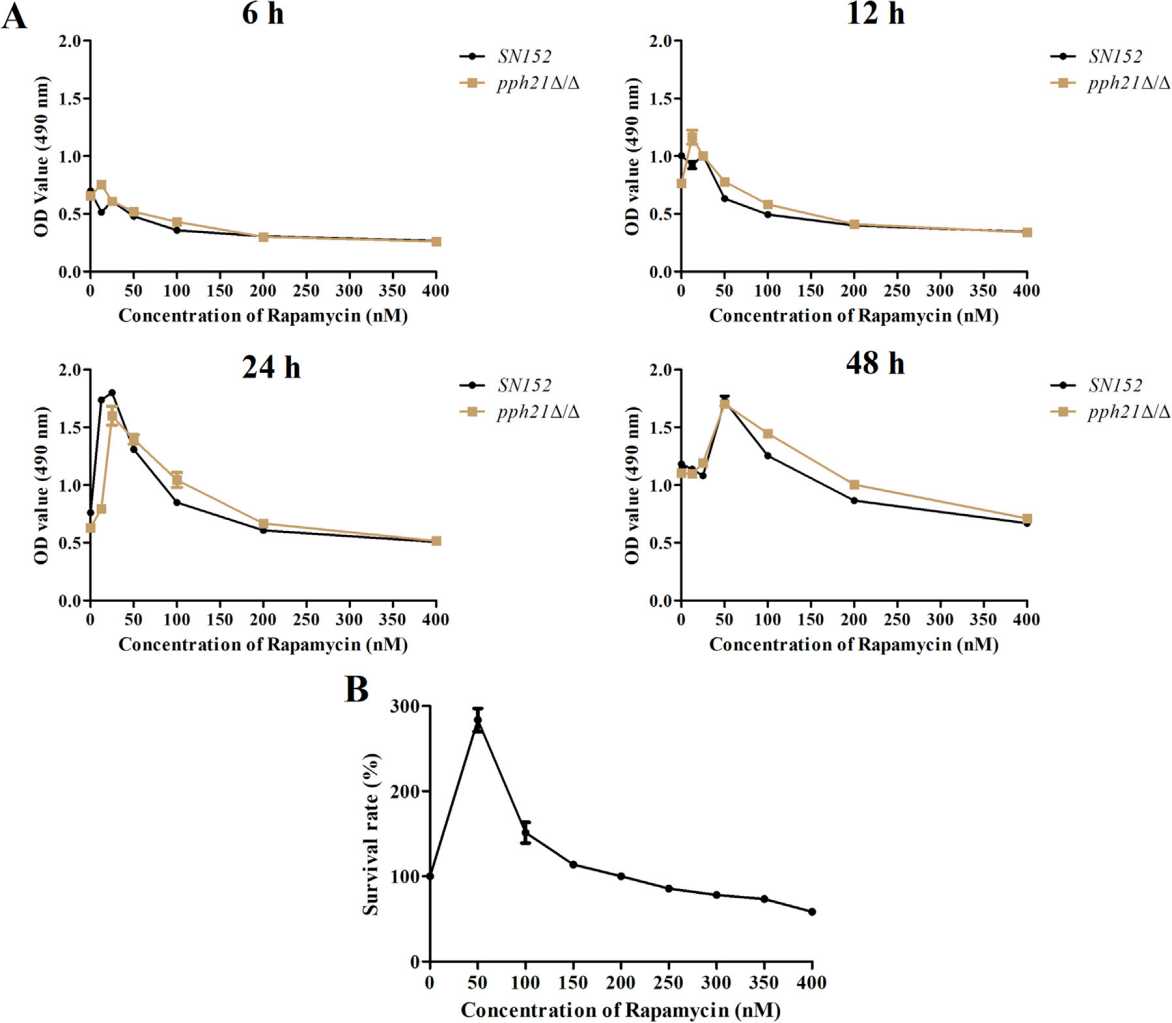
The biofilm formation of *C. albicans* after rapamycin treatment (0-400 nM). A, The comparison of biofilm formation between *SN152* and *pph21*Δ/Δ after treatment with rapamycin at different growth phases (6 h, 12 h, 24 h, and 48 h); B, The survival rate of *SN152* biofilm after rapamycin treatment for 24 h.

Biofilm formation of *C. albicans* is one of the methods to resist antifungal agents clinically.^43^ As the concentration of rapamycins increased (0-400 nM), the OD values of *pph21*Δ/Δ and *SN152* biofilm decreased at different growth phases (6 , 12 , 24 , and 48 h), indicating that high concentrations of rapamycin would limit the formation of *C. albicans* biofilm (Figure 2A). To acquire a suitable concentration of rapamycin that can induce autophagy, *SN152* was treated with different concentrations of rapamycin (0-400 nM) for 2 hours and the present study selected 200 nM and 100 nM rapamycin as the reference concentrations for subsequent experiments, where the biofilm survival rate of *SN152* was close to 100% at 200 nM (Figure 2B).

**Fig. 3:**
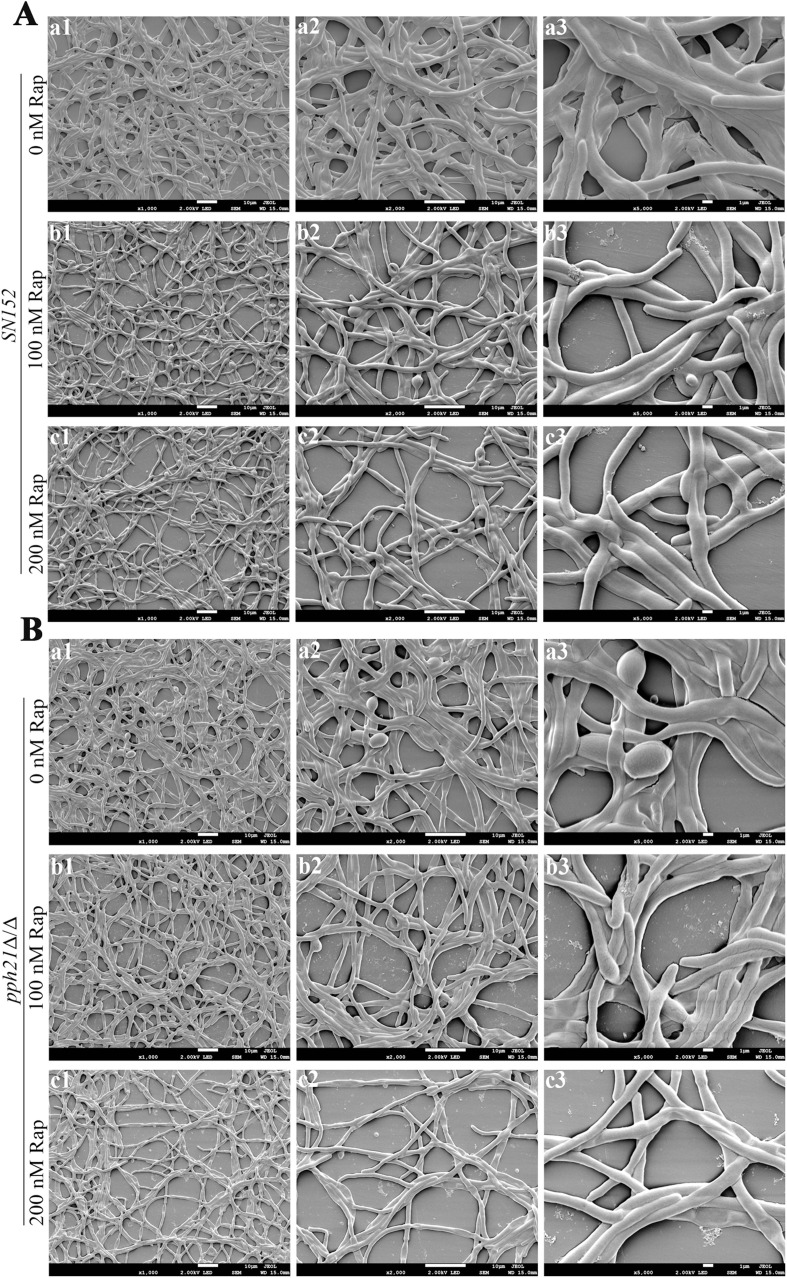
The morphological changes of the control group (*C. albicans SN152*) and *pph21*Δ/Δ biofilm treated with different concentrations of rapamycin via SEM. A, *SN152*; B, *pph21*Δ/Δ. a: 0 nM Rap; b: 100 nM Rap; c: 200 nM Rap. Magnification: 1: 1000 ×; 2: 2000 ×; 3: 5000 × .

Besides, the morphological structures of *C. albicans* biofilm were altered when exposed to rapamycin, manifested by a decrease in hyphal density (Figure 3); moreover, the ability to form hyphae decreased as the concentration of rapamycin increased, especially in *pph21*Δ/Δ (Figure 3B). In contrast, the untreated *SN152* and *pph21*Δ/Δ exhibited regular elongated hyphal forms, being smooth, dense, and well-structured. The above results suggested that autophagy of *C. albicans* may be involved in regulating biofilm formation.

### Deletion of *PPH21* altered the drug resistance of biofilm after autophagy activation by rapamycin

The present study investigated whether rapamycin altered the susceptibility of *C. albicans pph21*Δ/Δ to antifungal agents and the XTT assay results showed that SMIC_50_ of *pph21*Δ/Δ+Rap biofilm was lower compared to *SN152* without rapamycin treatment when treated with fluconazole (6 and 24 h), itraconazole (12 h), amphotericin B (48 h) and terbinafine (12 , 24 , and 48 h); however, *SN152*+Rap presented increased SMIC_50_ when treated with fluconazole (12 , 24 , and 48 h), amphotericin B (12 and 24 h), itraconazole and terbinafine (6 , 12 , 24 , and 48 h), indicating that autophagy activator rapamycin can enhance the drug resistance of *C. albicans* to some extent, while the deletion of *PPH21* may prevent the enhancement of drug resistance after autophagy activation (Table 3).

**Table 3:** The susceptibility of *SN152* and *pph21*Δ/Δ biofilm to antifungal agents (SMIC_50_ [mg/L]).

		SMIC_50_ of antifungal agents (mg/L)			
Strains		6 h	12 h	24 h	48 h
Fluconazole					
*SN152*		512	512	1024	1024
*SN152*+Rap		512	1024	>1024	>1024
*pph21*Δ/Δ		256	512	512	1024
*pph21*Δ/Δ+Rap		256	512	512	1024
Itraconazole					
*SN152*		256	512	1024	1024
*SN152*+Rap		512	1024	>1024	>1024
*pph21*Δ/Δ		256	512	512	512
*pph21*Δ/Δ+Rap		256	256	1024	1024
Amphotericin B					
*SN152*	2		2	2	4
*SN152*+Rap	2		4	4	4
*pph21*Δ/Δ	2		2	4	4
*pph21*Δ/Δ+Rap	2		2	2	2
Terbinafine					
*SN152*	256		512	1024	1024
*SN152*+Rap	512		1024	>1024	>1024
*pph21*Δ/Δ	256		256	512	1024
*pph21*Δ/Δ+Rap	256		128	512	512

Rap, rapamycin.

### Deletion of *PPH21* triggered increased oxidative stress after autophagy activation

**Fig. 4:**
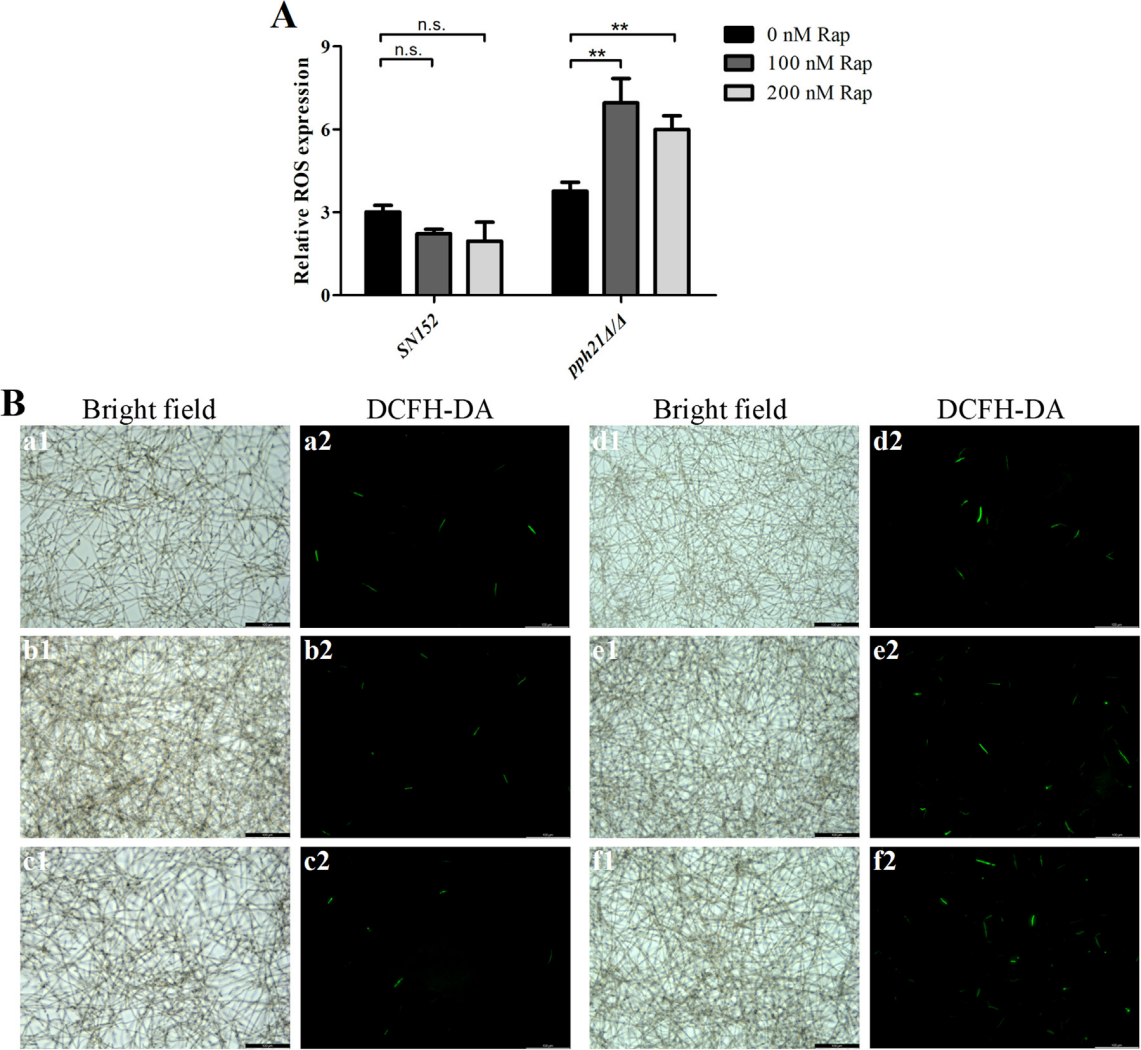
The comparison of ROS level between *SN152* and *pph21*Δ/Δ biofilm after rapamycin treatment. A, The ROS level of biofilm, ^⁎⁎^*P* < .01; n.s. no significant difference; B, Typical DCFH-DA fluorescence images of the biofilm, a. *SN152*; b. *SN152*+100 nM Rap; c. *SN152*+200 nM Rap; d. *pph21*Δ/Δ; e. *pph21*Δ/Δ+100 nM Rap; f. *pph21*Δ/Δ+200 nM Rap. Scale bar = 100 µm.

**Fig. 5:**
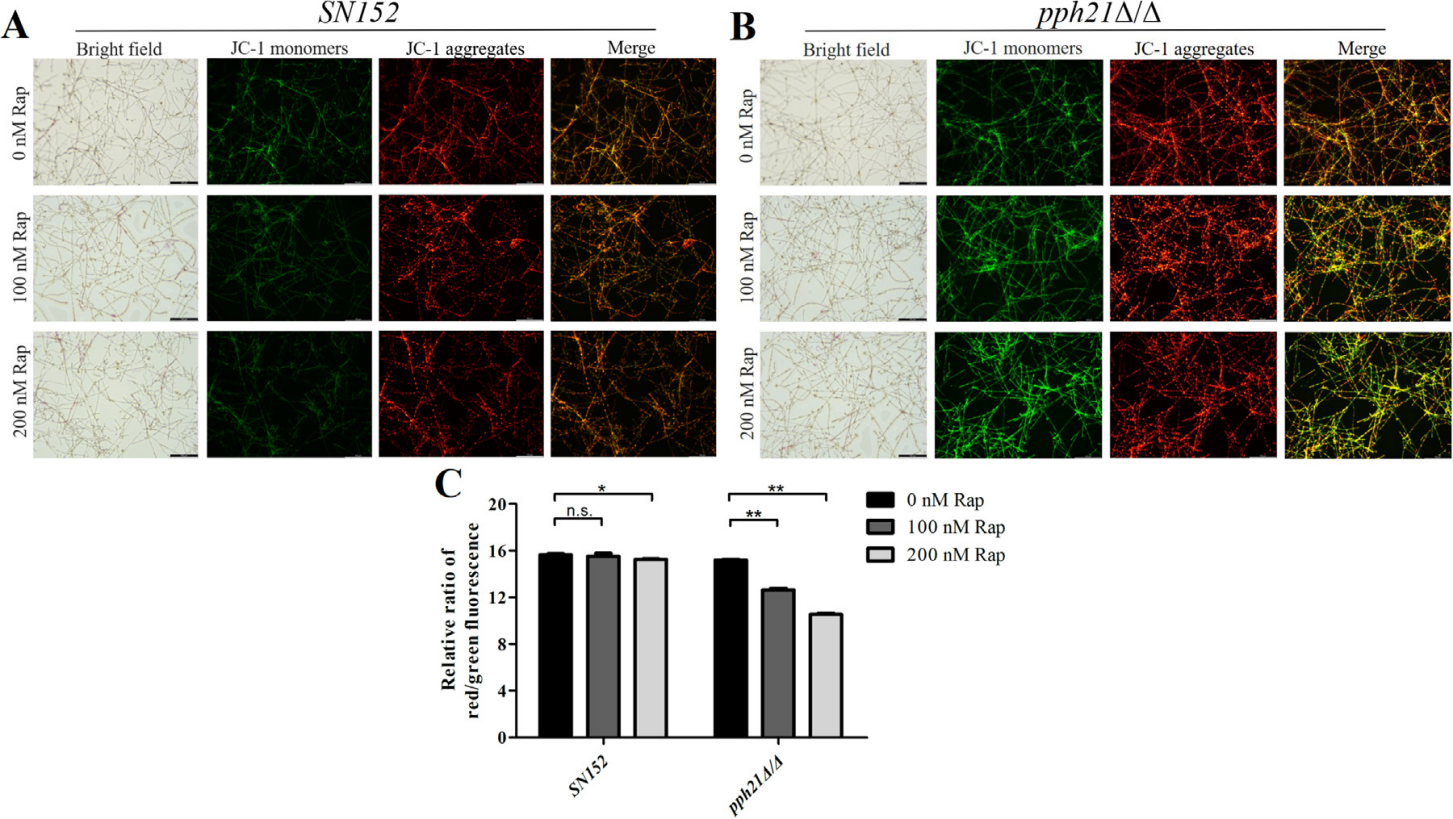
The comparison of MMP (ΔΨ) between *SN152* and *pph21*Δ/Δ after rapamycin treatment. A-B, Representative JC-1 fluorescence images of *SN152* and *pph21*Δ/Δ biofilm; scale bar = 100 µm. C, The relative ratio of JC-1 red/green fluorescence of the biofilm. *P < .05; ^⁎⁎^*P* < .01; n.s. no significant difference.

The ROS level of *pph21*Δ/Δ+Rap biofilm significantly increased (*P* < .01) than *pph21*Δ/Δ (Figure 4A), along with increased green fluorescence intensity detected by DCFH-DA (Figure 4B). Moreover, JC-1 aggregates (red fluorescence) in the mitochondrial matrix are converted into JC-1 monomers (green fluorescence) when ∆Ψm decreases. The green fluorescence intensity of *pph21*Δ/Δ+Rap was stronger than that of *pph21*Δ/Δ (Figure 5A-B), and concomitantly, increasing concentrations of Rap (0, 100, and 200 nM) triggered a dose-dependent reduction in the relative ratio of red/green fluorescence for both *SN152* and *pph21*Δ/Δ (especially in *pph21*Δ/Δ), with *pph21*Δ/Δ+Rap presenting significantly lower ratios compared to *pph21*Δ/Δ (*P* < .01) (Figure 5C). However, *SN152*+Rap did not exhibit enhanced ROS level or green fluorescence intensity (Fig. 4, Fig. 5), and there was no significant change (100 nM, *P* > .05) or decrease (200 nM, *P* < .05) in its relative ratio of red/green fluorescence compared with *SN152* (Figure 5C); hence, rapamycin treatment provoked increased oxidative stress of *pph21*Δ/Δ.

### Rapamycin-treatment failed to induce autophagy activation of *pph21*Δ/Δ

**Fig. 6:**
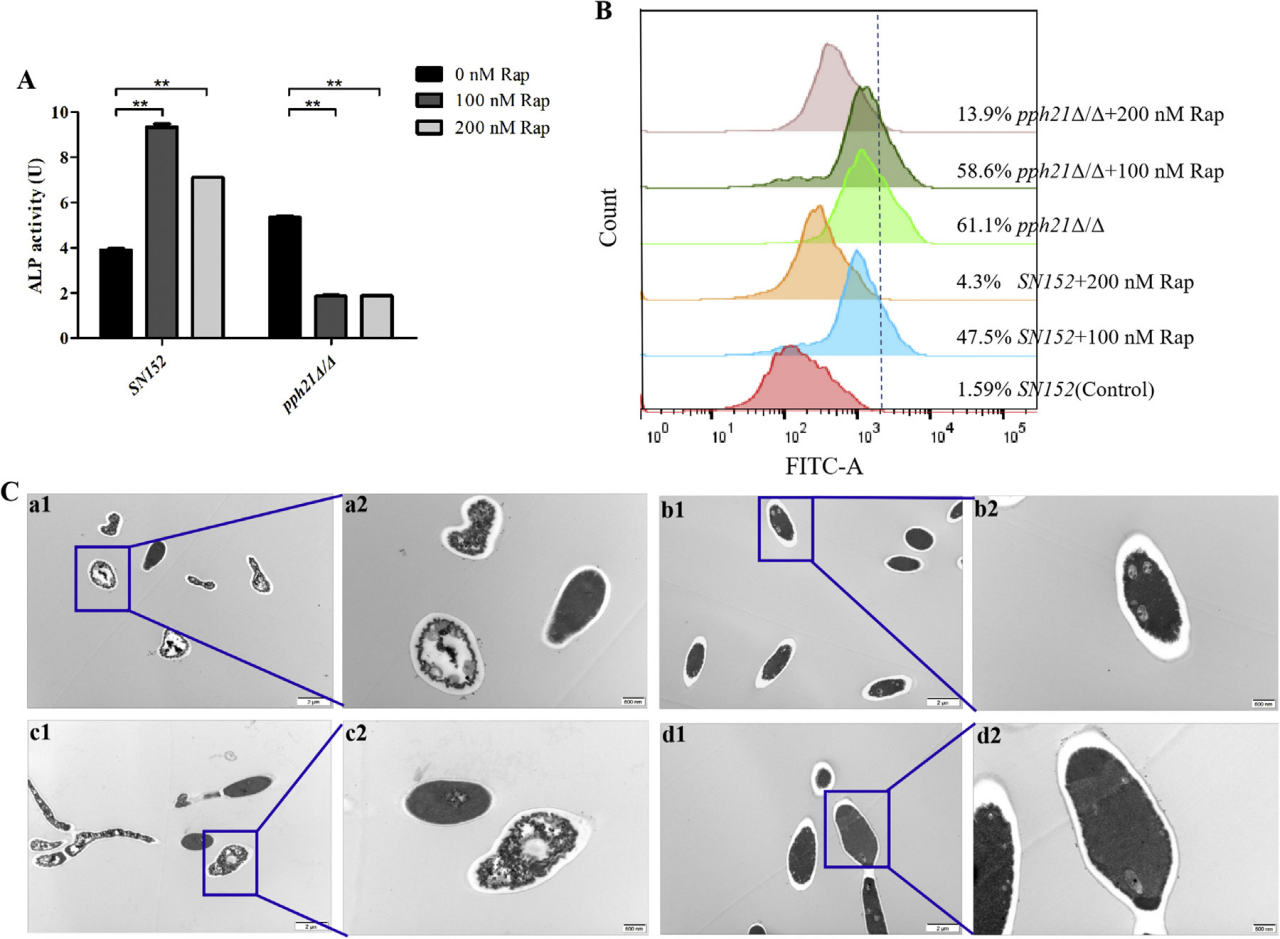
The autophagic activities of *SN152* and *pph21*Δ/Δ after rapamycin treatment. A, The ALP activity of the biofilm, ^⁎⁎^*P* < 0.01; B, The percentage of AO staining cells; C, The representative TEM images of the autophagosomes in biofilm. a. *SN152*; b. *SN152*+Rap; c. *pph21*Δ/Δ; d. *pph21*Δ/Δ+Rap. 1: scale bar = 2 μm; 2: scale bar = 500 nm.

The ALP activity of *pph21*Δ/Δ biofilm significantly decreased (*P* < .01) after rapamycin treatment, while it markedly increased for *SN152* (*P* < .01) (Figure 6A). Furthermore, the percentage of AO-positive cells (ie, acidic vesicular organelles, AVOs) was lower in *pph21*Δ/Δ+Rap than in *pph21*Δ/Δ, but it was higher in *SN152*+Rap than in *SN152* (Figure 6B). TEM showed that some cells of *pph21*Δ/Δ biofilm became elongated, with shrinkage in the cell walls, and no obvious autophagosomes were observed in *pph21*Δ/Δ+Rap biofilm cells, accompanied by partial dissolution and destruction of the contents. However, most of the biofilm cells in *SN152* and *SN152*+Rap exhibited normal morphology, manifested as round or spherical; besides, the autophagosomes, despite with undefined and indistinct cell membrane, were detected in *SN152*+Rap (Figure 6C), indicating that rapamycin can activate autophagy and induce the increased autophagic activities of *C. albicans*, while it failed to induce autophagy activation of *pph21*Δ/Δ.

### *PPH21* was required for sufficient Atg13 dephosphorylation and Atg1 activation when autophagy was activated

**Fig. 7:**
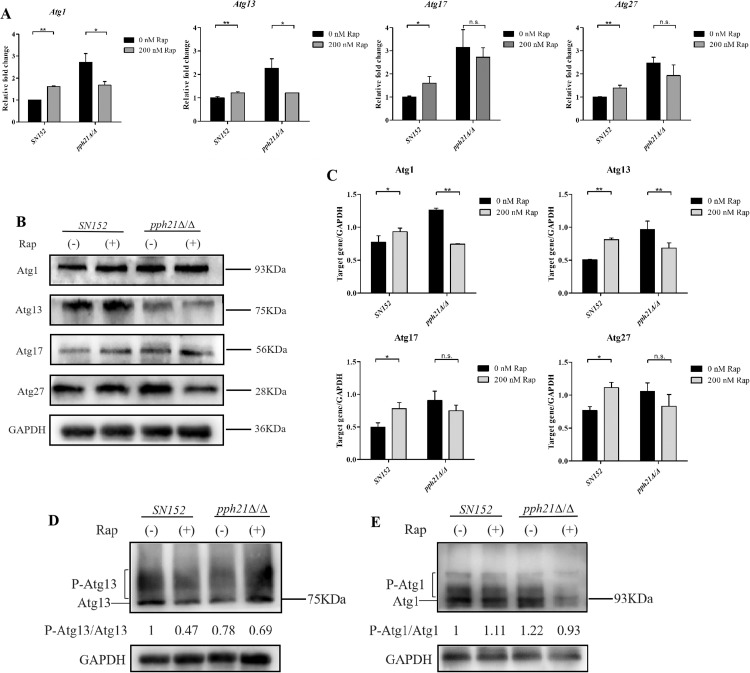
The expressions of autophagy-related genes and proteins in *SN152* and *pph21*Δ/Δ biofilm after the activation of autophagy. A, Expressions of autophagy-related genes (*Atg1, Atg13, Atg17*, and *Atg27*); B, Protein expression levels of Atg1, Atg13, Atg17, and Atg27; C, Quantitative protein analysis of western blotting from (B); *P < .05; ^⁎⁎^*P* < .01; n.s. no significant difference. D, The ratio of phosphorylated Atg13 and dephosphorylated Atg13. P-Atg13, phosphorylated Atg13; E, The ratio of phosphorylated Atg1 and dephosphorylated Atg1. P-Atg1, phosphorylated Atg1. Relative values normalized against the value in *SN152* without rapamycin treatment were shown.

To explore the possible mechanism underlying the differences in autophagy activation between *SN152* and *pph21*Δ/Δ, the researches regarding the autophagy signalling pathway were subsequently conducted. After rapamycin treatment, the expressions of autophagy-related genes (*Atg1, Atg13, Atg17*, and *Atg27*) in *SN152* biofilm significantly increased (*P* < .05). In contrast, the expressions of *Atg1* and *Atg13* in *pph21*Δ/Δ significantly decreased (*P* < .05), accompanied by no significant changes in *Atg17* and *Atg27*, supposed that *Atg1* and *Atg13* may play important roles in the activation of autophagy in *C. albicans* (Figure 7A). The above finding was further confirmed by the protein expression levels and quantitative protein analysis of western blotting (Figure 7B-C), indicating that Atg13 and Atg1 are key proteins in the autophagy signalling pathway of *C. albicans*.

Atg13 is hyperphosphorylated under normal nutrient conditions and dephosphorylated after rapamycin treatment, manifested as a change in the ratio of phosphorylated Atg13 and dephosphorylated Atg13 (P-Atg13/Atg13). The ratio of P-Atg13/Atg13 in *pph21*Δ/Δ was higher than that in *SN152*; however, it decreased in *SN152* due to the dephosphorylation of Atg13, suggesting that the deletion of *PPH21* may partially impede the dephosphorylation of Atg13 upon rapamycin treatment, ultimately causing incomplete activation of autophagy (Figure 7D). In addition, the slower-migrating band (activated Atg1) was relatively obvious in *SN152*+Rap, while it was partially repressed in *pph21*Δ/Δ+Rap; moreover, the ratio of P-Atg1/Atg1 was lower in *pph21*Δ/Δ+Rap compared to *SN152* (Figure 7E), implying that the activation of Atg1 in *pph21*Δ/Δ+Rap was also partially inhibited, which may be attributed to the reduced protein levels and insufficient dephosphorylation of Atg13 in *pph21*Δ/Δ+Rap, further impairing Atg1 activation.

### Deletion of *PPH21* affected the therapeutic effects of antifungal agents on *C. albicans*-infected mice

**Fig. 8:**
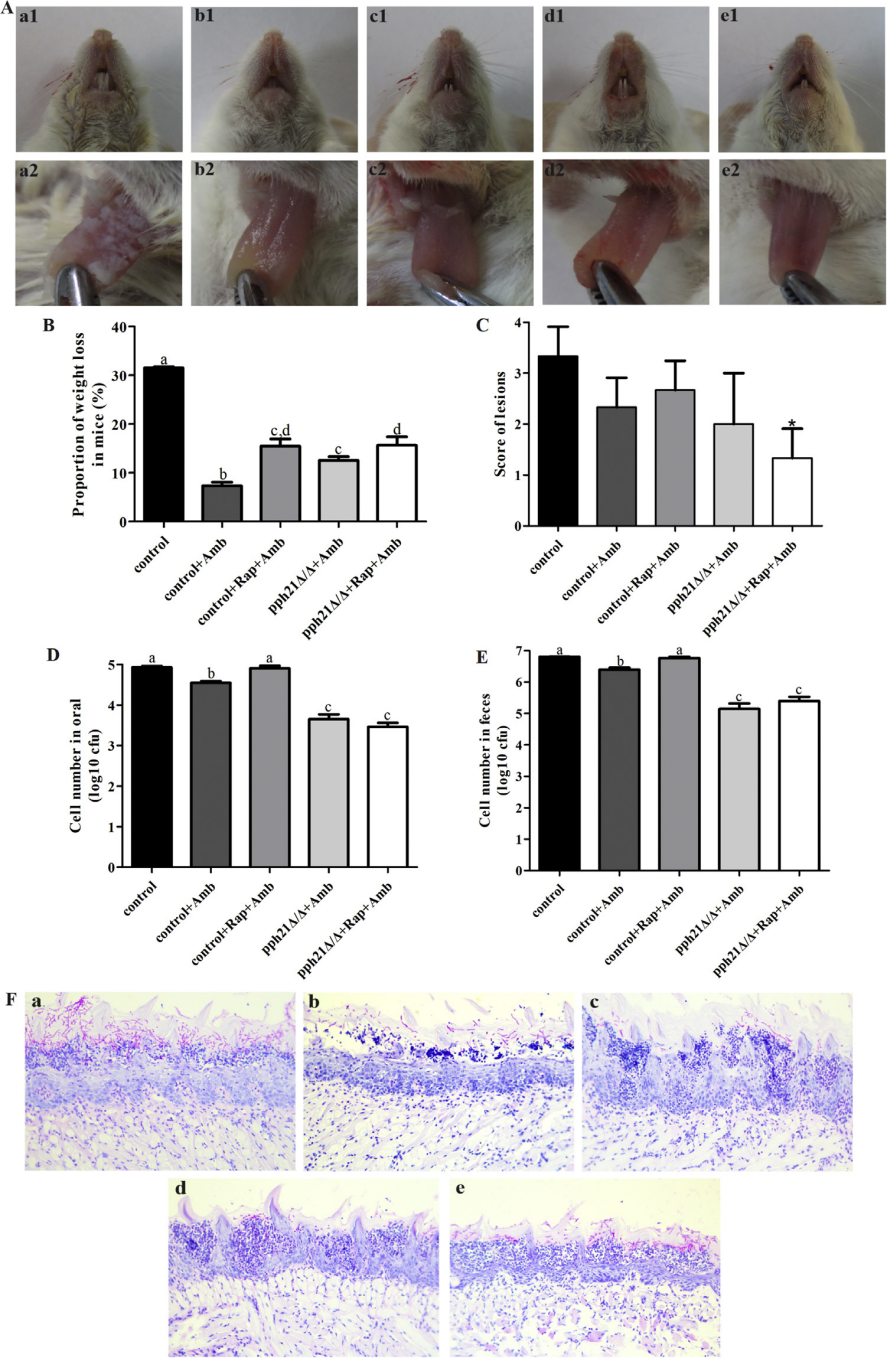
Autophagy activation affected the therapeutic effects of antifungal agents on oral infections caused by *C. albicans* in mice. A, Macroscopic observation of white pseudomembranes around the mouth and tongue of mice with oral candidiasis; B, Proportion of weight loss in mice (%); C, Score of tongue lesions; D-E, The number of live cells of *C. albicans* in oral D, or in feces E; values labeled with the same superscript are not significantly different (*P* > .05). F, Histopathological observation of a longitudinal section of the tongue of the mice (× 200 magnification). The tissue sections were stained with PAS stain. a. control (*SN152*); b. control+Amb; c. control+Rap+Amb; d. *pph21*Δ/Δ+Amb; e. *pph21*Δ/Δ+Rap+Amb.

The murine oral candidiasis model was successfully constructed according to a previous study,^41^ and this study chose the 3rd day as the observation time point for subsequent experiments *in vivo*.^42^ The antifungal agent (amphotericin B, Amb) presented a certain therapeutic effect on *C. albicans-*caused infections in the control (*SN152*) and *pph21*Δ/Δ mice. Compared to the control, the depilation around the mouth in mice treated with Amb improved, along with reduced pseudomembranes on the tongue (Figure 8A) and proportion of weight loss (Figure 8B); furthermore, the score of tongue lesions also decreased, among which *pph21*Δ/Δ+Rap+Amb was the lowest (Figure 8C). Additionally, the number of CFU in oral and feces in the Amb-treated mice decreased, lower in *pph21*Δ/Δ+Amb and *pph21*Δ/Δ+Rap+Amb than in other groups; however, it did not show a prominent reduction in control+Rap+Amb compared with the control (*P* > .05) (Figure 8D-E). PAS-positive *C. albicans* was observed inside the oral epithelial layer under the microscope. Treatments with Amb reduced the amount of *C. albicans* hyphae and inflammatory cells in the epithelia of the tongue in the infected mice (Figure 8F). Nevertheless, the proportion of weight loss and score of tongue lesions in control+Rap+Amb were higher than those of control+Amb (Figure 8B-C), indicating that autophagy activation may reduce the efficacy of antifungal agents in treating oral candidiasis to some extent, which may be owing to its enhanced pathogenicity of *C. albicans*.

## Discussion

Autophagy is a conserved catabolic process that is necessary to circumvent nutrient deficiency and maintain cellular homeostasis.^44^^,^^45^ Previous studies have reported that the expressions of some autophagy-related genes in *C. albicans* biofilm were significantly increased, indicating that autophagy was associated with biofilm formation of *C. albicans*.^46^ However, so far, there is little research on the regulation of autophagy on biofilm formation and drug resistance in *C. albicans*, and the molecular mechanisms still need to be elucidated. This study applied rapamycin as an autophagy activator to explore the possible mechanism by which PP2A regulates autophagy and affects biofilm formation and susceptibility to antifungals.

ALP activity and AO staining are reliable methods for evaluating autophagic activities, usually used to characterize changes in autophagy levels.^47^ The ALP activity (*P* < .01) and the percentage of AO-positive cells of *pph21*Δ/Δ decreased after rapamycin treatment, along with no obvious autophagosomes detected; meanwhile, the autophagic activities of *SN152* increased, indicating that rapamycin can activate autophagy of *C. albicans* and induce increased autophagy levels; however, the autophagy activation of *pph21*Δ/Δ is hindered, leading to a decrease in autophagic activities.

Atg13 dephosphorylation is a critical event in triggering the induction of autophagy after TORC1 inhibition,^48^ that allows Atg13 to interact with Atg1, followed by the upregulation of the kinase activity of Atg1 and forms the Atg1 kinase complex, consequently leading to a change in the ratio of P-Atg13/Atg13, as well as P-Atg1/Atg1. The absence of *PPH21* partially impeded the dephosphorylation of Atg13, resulting in incomplete activation of autophagy of *C. albicans*. Furthermore, the reduced protein levels of Atg13 and insufficient dephosphorylation of Atg13 in *pph21*Δ/Δ after rapamycin treatment may eventually compromise Atg1 activation. The interaction of Atg13 dephosphorylation to Atg1 is necessary for the activation of Atg1 and subsequent induction of autophagy.^49^ Accordingly, *PPH21* was involved in sufficient dephosphorylation of Atg13 followed by Atg1 activation after the inactivation of TORC1 in *C. albicans* and may be located downstream of TORC1, affecting its regulatory effect on autophagy. A previous research has found that TORC1 affects the activity of PP2A in *Saccharomyces cerevisiae* (*S. cerevisiae*), thereby influencing autophagy induction,^50^ which was also confirmed by the present study. However, it was noted that Atg13 dephosphorylation can still occur in *pph21*Δ/Δ when TORC1 was inhibited, indicating that there may be additional protein phosphatases involved in dephosphorylation of Atg13 in *C. albicans*. Besides, several phosphatases may synergistically participate in Atg13 dephosphorylation and autophagy induction, which may also explain why rapamycin can still promote the biofilm formation of *pph21*Δ/Δ to some extent. Multiple phosphorylation sites exist on Atg13;^51^ however, how they participate in regulating the formation of Atg1 complexes and autophagy induction remains unknown, and the related molecular mechanisms need to be further explored.

The oxidative stress of *pph21*Δ/Δ increased after rapamycin treatment, manifested as an increase in ROS levels and a decrease in MMP, which may be due to incomplete activation of autophagy in *pph21*Δ/Δ, resulting in decreased autophagy levels and reduced its ability to regulate oxidative stress. Conversely, the autophagy levels of *SN152* increased, and its capacity to regulate oxidative stress was enhanced; thus, its oxidative stress did not significantly increase caused by rapamycin treatment. Although autophagic activities mostly occur at basic levels,^52^ they can also be upregulated under various stress conditions, such as hypoxia, oxidative stress, and pathogen infection, especially under nutrient starvation.^53^^,^^54^

ROS production might be profitable to inhibit fungal growth and reduce the drug resistance of fungi.^55^ In addition, *PPH21* and *PPH22* genes each contribute approximately half of the PP2A activity, and deletion of either gene has no notable effect on cell growth; however, deletion of both genes will eliminate 80% to 90% of PP2A activity, which profoundly hinders cell growth.^56^ In this study, the deletion of *PPH21* may also account for affecting the biofilm formation and drug resistance. High concentrations of rapamycin can limit the biofilm formation of *C. albicans*, indicating that autophagy is relevant to regulating the biofilm formation of *C. albicans*. Autophagy activation can also enhance the drug resistance of *C. albicans*, while *pph21*Δ/Δ presented no significant increase in resistance to antifungal agents. This may be attributed to the incomplete activation of autophagy in *pph21*Δ/Δ after rapamycin treatment, which reduced the regulatory effect of autophagy on its oxidative stress, ultimately leading to its increased oxidative stress and impeding the enhancement of resistance.

Additionally, *in vivo* experiments showed that antifungal agents can alleviate the oral infection caused by *C. albicans* in mice, while autophagy activation reduced its efficacy in treating oral infection, which may be related to the enhanced pathogenicity of *C. albicans* by autophagy activation. Among them, the oral infection of *pph21*Δ/Δ+Rap+Amb was relatively mild, which was consistent with the *in vitro* experiments that the autophagy activation of *pph21*Δ/Δ was blocked, leading to reduced drug resistance and weakened pathogenicity, further improving the therapeutic efficacy of Amb on the infected mice.

In summary, autophagy is one of the regulatory mechanisms for biofilm formation and drug resistance of *C. albicans*. This study provided a theoretical basis for the molecular mechanism of PP2A regulating autophagy of *C. albicans*. PP2A participates in the dephosphorylation of Atg13 under starvation conditions or after treatment with rapamycin, and the interaction between dephosphorylated Atg13 and Atg1 is essential for the activation of Atg1, which is conducive to further inducing autophagy of *C. albicans*. Nevertheless, autophagy activation may reduce the efficacy of antifungal agents in oral candidiasis treatment.

## Conclusion

The present study induced autophagy with the application of an autophagy activator (rapamycin) and revealed the molecular mechanism by which PP2A regulates autophagy through ATG phosphorylation, thereby affecting the biofilm formation and drug resistance of *C. albicans*. Hence, regulation of autophagy may be a potential therapeutic strategy for treating *C. albicans*-related infectious diseases and contributing to improving the susceptibility to clinical antifungal agents of *C. albicans*.

## Conflict of interest

None disclosed.
